# Dual sEH/COX-2 Inhibition Using PTUPB—A Promising Approach to Antiangiogenesis-Induced Nephrotoxicity

**DOI:** 10.3389/fphar.2021.744776

**Published:** 2021-12-09

**Authors:** Wojciech K. Jankiewicz, Scott D. Barnett, Anna Stavniichuk, Sung Hee Hwang, Bruce D. Hammock, Jawad B. Belayet, A. H. Khan, John D. Imig

**Affiliations:** ^1^ Drug Discovery Center and Cardiovascular Center, Medical College of Wisconsin, Milwaukee, WI, United States; ^2^ Department of Entomology and Nematology and Comprehensive Cancer Center, University of California, Davis, Davis, CA, United States; ^3^ Department of Chemistry and Biochemistry, University of Wisconsin Milwaukee, Milwaukee, WI, United States

**Keywords:** cyclooxygenase (COX), soluble epoxide hydrolase (sEH), vascular endothelial growth factor, nephrotoxicity, kidney injury, glomerular injury, eicosanoids, multitarget drugs

## Abstract

Kidney injury from antiangiogenic chemotherapy is a significant clinical challenge, and we currently lack the ability to effectively treat it with pharmacological agents. Thus, we set out to investigate whether simultaneous soluble epoxide hydrolase (sEH) and cyclooxygenase-2 (COX-2) inhibition using a dual sEH/COX-2 inhibitor PTUPB could be an effective strategy for treating antiangiogenic therapy-induced kidney damage. We used a multikinase inhibitor, sorafenib, which is known to cause serious renal side effects. The drug was administered to male Sprague–Dawley rats that were on a high-salt diet. Sorafenib was administered over the course of 56 days. The study included three experimental groups; 1) control group (naïve rats), 2) sorafenib group [rats treated with sorafenib only (20 mg/kg/day p.o.)], and 3) sorafenib + PTUPB group (rats treated with sorafenib only for the initial 28 days and subsequently coadministered PTUPB (10 mg/kg/day i.p.) from days 28 through 56). Blood pressure was measured every 2 weeks. After 28 days, sorafenib-treated rats developed hypertension (161 ± 4 mmHg). Over the remainder of the study, sorafenib treatment resulted in a further elevation in blood pressure through day 56 (200 ± 7 mmHg). PTUPB treatment attenuated the sorafenib-induced blood pressure elevation and by day 56, blood pressure was 159 ± 4 mmHg. Urine was collected every 2 weeks for biochemical analysis. After 28 days, sorafenib rats developed pronounced proteinuria (9.7 ± 0.2 P/C), which intensified significantly (35.8 ± 3.5 P/C) by the end of day 56 compared with control (2.6 ± 0.4 P/C). PTUPB mitigated sorafenib-induced proteinuria, and by day 56, it reduced proteinuria by 73%. Plasma and kidney tissues were collected on day 56. Kidney histopathology revealed intratubular cast formation, interstitial fibrosis, glomerular injury, and glomerular nephrin loss at day 56 in sorafenib-treated rats. PTUPB treatment reduced histological features by 30%–70% compared with the sorafenib-treated group and restored glomerular nephrin levels. Furthermore, PTUPB also acted on the glomerular permeability barrier by decreasing angiotensin-II-induced glomerular permeability to albumin. Finally, PTUPB improved *in vitro* the viability of human mesangial cells*.* Collectively, our data demonstrate the potential of using PTUPB or dual sEH/COX-2 inhibition as a therapeutic strategy against sorafenib-induced glomerular nephrotoxicity.

## Introduction

Antiangiogenic drugs are widely used in cancer treatment. They block neovascularization of tumors and, thus, prevent tumor growth. Vascular endothelial growth factor (VEGF) tyrosine kinase inhibitors (TKIs) are a major class of these drugs. They are used to treat malignant neoplasms and, more recently, age-related neovascular macular degeneration, which is an irreversible eye disease leading to blindness (Apte et al., 2019). VEGF TKIs comprise a wide class of compounds: sorafenib, regorafenib, axitinib, cabozantinib, lenvatinib, nintedanib, pazopanib, sunitinib, and vandetanib. To date, the FDA has approved sorafenib for three indications: treatment of advanced renal cell carcinoma (approved in 2005), treatment of inoperable hepatocellular carcinoma (approved in 2007), and treatment of metastatic differentiated thyroid cancer (approved in 2013) (White and Cohen, 2015). Sorafenib inhibits angiogenesis by targeting c-Kit, FLT-3, VEGFR-2, VEGFR-3, and PDGFR-β, and inhibits proliferation through targeting Raf-1, B-Raf, and Ras/Raf/MEK/ERK (Zhu et al., 2017). VEGF TKIs are used alone or in combination therapy with immune checkpoint inhibitors (Rassy et al., 2020). With around 400,000 cases of renal cell carcinoma, 500,000 cases of hepatocellular carcinoma, and 500,000 cases of thyroid cancer diagnosed each year, to date, sorafenib has potentially saved thousands of lives (Bray et al., 2018).

Unfortunately, VEGF TKIs come with severe limitations in the form of hypertension, proteinuria, and renal injury (Humphreys and Atkins, 2009; Jhaveri et al., 2011; Estrada et al., 2019; Versmissen et al., 2019; Neves et al., 2020). Current guidelines involve monitoring and management of the side effects including blood pressure medications to lower hypertension (Versmissen et al., 2019). Renal injury from VEGF TKIs includes glomerular barrier breakdown, mesangiolysis, and thrombotic microangiography (Kelly et al., 2009; Overkleeft et al., 2009; Estrada et al., 2019). These are serious problems because kidney damage can force discontinuation of an otherwise effective anticancer therapy as glomerular injury can progress to chronic kidney disease or life-threatening end-stage renal disease (Venkatachalam et al., 2010). Importantly, there are no pharmacological means that could help protect the kidneys from the injury. A strong need exists for a pharmacological agent that could diminish kidney injury that is caused by VEGF TKIs. We propose that dual soluble epoxide hydrolase (sEH)/cyclooxygenase-2 (COX-2) inhibition can protect the kidneys from VEGF-TKI-induced damage by decreasing glomerular damage.

PTUPB is a dual sEH/COX-2 inhibitor, which acts to increase epoxyeicosatrienoic acids and decrease COX-2 inflammatory prostanoids (Cheng et al., 2002; Hye Khan et al., 2016; Sun et al., 2020). sEH inhibition lowers blood pressure, decreases inflammation, and can combat glomerular and kidney injury (Yu et al., 2000; Imig, 2012; Kim et al., 2014; Liu, 2019; Jiang et al., 2020). COX-2 is involved in the production of inflammatory prostanoids, and its inhibition is known to decrease kidney injury (Cheng et al., 2002; Fujihara et al., 2003; Harris, 2013). Earlier studies have shown PTUPB to highly selectively inhibit COX-2 over COX-1 (Hwang et al., 2011).

Our lab has previously shown that PTUPB can mitigate kidney injury in a rat model of diabetic nephropathy (Hye Khan et al., 2016). Other studies have shown that PTUPB can reduce inflammation, oxidative stress, and cell senescence (Dileepan et al., 2019; Sun et al., 2020; Zhang et al., 2020). PTUPB has also been reported to improve nonalcoholic fatty liver disease, at least in part, through inhibiting inflammation (Sun et al., 2020). Previous work also indicates that PTUPB can prevent cisplatin-, carboplatin-, and paclitaxel-induced cytokine and eicosanoid storm and suppress debris-stimulated ovarian tumor growth (Zhang et al., 2020). Emerging evidence also suggests that PTUPB has antitumor activity and can potentiate tumor cytotoxicity of other drugs (Li et al., 2017; Wang et al., 2018). In the current study, we demonstrate that interventional PTUPB treatment can protect the kidney from sorafenib-induced nephrotoxicity.

## Materials and methods

### Chemicals

The chemistry and synthesis process of dual COX-2/sEH inhibitor, 4-(5-phenyl-3-{3-[3-(4-trifluoromethylphenyl)-ureido]-propyl}-pyrazol-1-yl)-benzenesulfonamide (PTUPB) was described earlier (Hwang et al., 2011). Sorafenib was obtained from LC Laboratories (Woburn, MA, USA). Unless otherwise stated, all chemicals used in this study were obtained from Sigma Aldrich (St. Louis, MO, USA).

### Animal study

**FIGURE 1:**
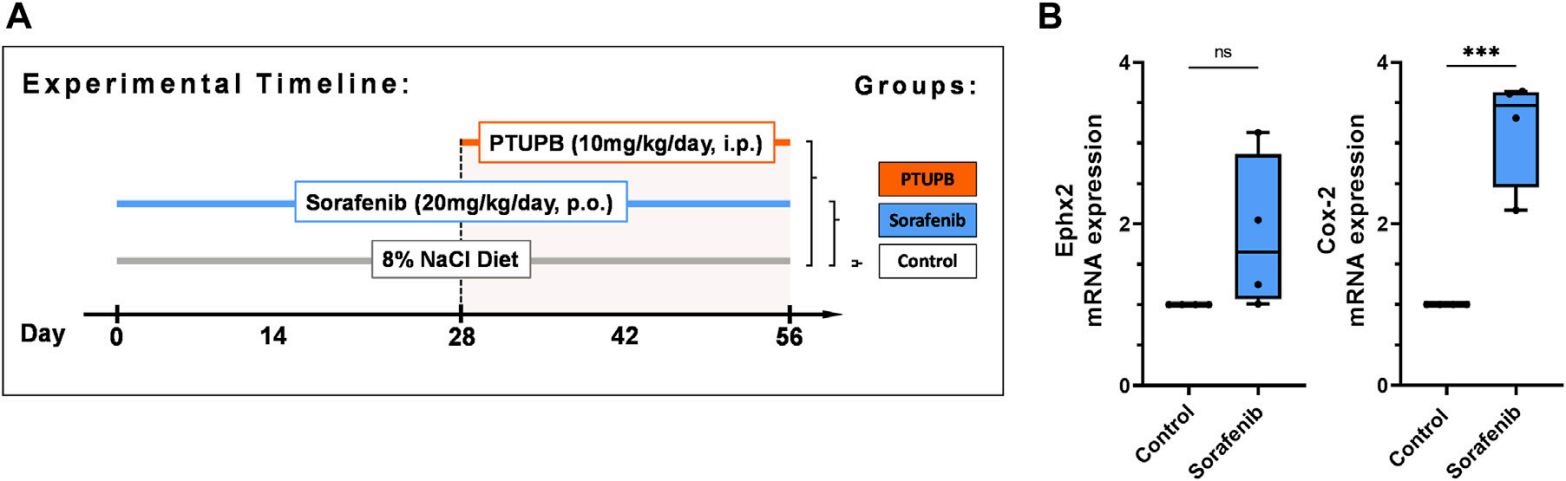
Overview. Timeline showing the experimental design **(A)**. Expression of soluble epoxide hydrolase enzyme (Ephx2) and cycolooxygenase-2 (COX-2) sorafenib treatment **(B)**. (*n* = 4–6 rats/group, Dots represent the average of duplicate measures for each individual rat. ns *p* > 0.05, ***p ≤ 0.001 determined by Student’s t-test. Data are reported as box and whisker plots with median, minimum to maximum, and 10 to 90 percentiles.

This study was approved and carried out according to the guidelines of the Medical College of Wisconsin Institutional Animal Care and Use Committee. The Biomedical Resource Center at the Medical College of Wisconsin housed animals with free access to water and food under a 12/12 h light–dark cycle. Male Sprague–Dawley rats (8–10 weeks old) were purchased from Charles River Laboratories, Spokane, IL, USA. The study comprised three treatment groups: control group, sorafenib group, and sorafenib + PTUPB group (Figure 1A, *n* = 8 rats/group). Rats were acclimated to blood pressure measurements over the course of 7 days prior to the commencement of the study. The animals were placed on a high-salt diet (8% NaCl) (TD.92012**,** Envigo, Madison, WI, USA). Systolic blood pressure was measured on days 0, 14, 28, 42, and 56 using a tail cuff system (IITC Life Science, Woodland Hills, CA, USA). Sorafenib was orally administered to the sorafenib and sorafenib + PTUPB groups at a dose of 20 mg/kg/day. PTUPB was coadministered in the sorafenib + PTUPB group at a dose of 10 mg/kg/day from day 28 through 56 through an intraperitoneal osmotic pump (ALZET® osmotic pump, DURECT Corporation, Cupertino, CA, USA). Urine samples were collected using metabolic cages on days 0, 28, and 56. On day 56, animals were euthanized; blood and kidney samples were obtained. Kidney samples for histological and immunohistochemical studies were fixed in 10% buffered formalin and stored at room temperature. Kidney tissue samples for gene expression analysis were snap frozen in liquid nitrogen and stored at −80°C.

### Real-time polymerase chain reaction

MRNA expression of Ephx2, Cox-2, ZEB1, TWIST, and α-SMA was determined by real-time polymerase chain reaction (RT-PCR). Samples were homogenized using TissueLyser II (Qiagen, Redwood City, CA, USA). RNA was extracted from sample homogenates using the RNeasy Mini Kit (Qiagen, Redwood City, CA, USA) according to the protocol of the manufacturer. The RNA samples were quantified spectrophotometrically with a NanoDrop, and 1 μg of total RNA was reverse transcribed to cDNA using iScript™ Select cDNA Synthesis Kit (Bio-Rad, Hercules, CA, USA). Gene expression was quantified by iScript One-Step RT-PCR Kit with SYBR green using the MyiQ™ Single Color RT-PCR Detection System (Bio-Rad Laboratories, Hercules, CA, USA). Dissociation curve analysis was performed with iQ5 Optical System Software, Version 2.1 (Bio-Rad Laboratories, Hercules, CA, USA). Each amplified sample was analyzed for homogeneity. Samples were denatured at 95°C for 2 min. Next, the PCR was performed using a protocol of 40 cycles at 95°C for 10 s and at 60°C for 30 s. Samples were run in triplicate. Gene expression fold changes were compared with controls determined by the comparative threshold cycle (Ct) method. Target gene expression levels were determined by normalizing Ct values to housekeeping genes. Statistical analyses were carried out using six samples from each experimental group and comparing with the control group.

### Histology

Renal tissues were fixed in 10% formalin, sectioned at 5-μm thickness, mounted on slides, and stained with periodic acid-Schiff (PAS) (Acros Organics, Fairlawn, NJ, USA) or picrosirius red (PSR) (Alfa Aesar, Tewksbury, MA, USA). PAS-stained renal sections were evaluated for the presence of tubular casts. PSR-stained renal sections were evaluated for collagen-positive renal interstitial fibrotic changes and expressed as percent area relative to the total area analyzed. Glomerular injury was blindly scored on kidney sections stained with PAS staining using the following numeric scale: 0 = no damage; +1 = very mild; +2 = mild; +3 = moderate and +4 = severe. All analyses were conducted by two observers in a blinded fashion for histological examination at ×200 magnification using NIS Elements AR version 3.0 imaging software (Nikon Instruments Inc., Melville, NY, USA).

### Immunofluorescence

Kidney slides were deparaffinized and rehydrated followed by overnight incubation with an anti-nephrin antibody (1:100; Santa Cruz Biotechnology, Inc., Dallas, TX, USA) to determine renal expression of nephrin. Donkey anti-rabbit IgG H&L (Alexa Fluor® 488) secondary antibody (1:200; Abcam, Cambridge, MA, USA) was used for development with fluorescence quenching liquid (Vector Laboratories, Burlingame, CA, USA). Stained histological sections were examined with a Nikon 55i microscope at ×200 magnification with fluorescent excitation, and images were analyzed using Nikon NIS Elements Software (Nikon Instruments Inc., Melville, NY, USA). Positively stained areas specific for the target protein used were expressed as percent area relative to total area analyzed. Analyses were carried out by two observers blinded to sample identity.

### Glomerular permeability

Glomeruli were isolated from adult male Sprague–Dawley rats, and the experiment was performed following a previously described protocol (Ilatovskaya et al., 2017). First, to label the inside of the glomeruli, kidneys were perfused through the femoral vein with an FTIC–dextran solution (150-kDa FTIC–dextran in 0.9% NaCl) (TdB Consultancy AB, Uppsala, Sweden) and isolated. The following steps were performed on ice: The kidney cortex was separated from the medulla and cut into 1-mm^3^ cubes. Next, the glomeruli were separated by differential sieving (sieve nos. 100, 150, and 200). Sieve no. 200 was used to capture the glomeruli. The isolated glomeruli were stored in a 5% BSA–TRITC–dextran solution (150-kDa TRITC–dextran and 5% BSA in RMPI) (TdB Consultancy AB, Uppsala, Sweden) and stored on ice for immediate use in experiments. The isolated glomeruli were incubated with angiotensin II (002-12, Phoenix Pharmaceuticals) for 30 min, and co-incubated with angiotensin II and PTUPB for 30 min. Under experimental conditions, the 5% BSA bath solution was exchanged for a 1% BSA solution, and a change in glomerular volume occurred due to the oncotic gradient. Glomerular volume changes were monitored using the Nikon A1R+ (Nikon Instruments Inc., Melville, NY, USA) and calculated from z-stack reconstructions using the Fiji image analysis software (ImageJ 1.52s, National Institute of Health, MD, USA) (Schindelin et al., 2012). The relative change in volume, {ΔV = [(V_final_ − V_initial_)/V_initial_] * 100}, can be compared with control values to obtain the ratio of the oncotic force exerted by that solute to its theoretical oncotic force, which is called the reflection coefficient (σalb = ΔV_experimental_/ΔV_control_). From here a conventional permeability value is obtained (Palb = 1 − σalb) for which a value of “1” denotes complete permeability of albumin, and a value of “0” signifies no permeability of albumin relative to the control.

### Cell culture

Human renal mesangial cells (4200, ScienCell, Carlsbad, CA, USA) were cultured at 37°C (in 5% CO_2_) in RPMI 1640 medium (Gibco™, LS11875093) containing 10% FBS, 100 U/ml of penicillin, and 0.1 mg/ml of streptomycin. The cells were subcultured following the protocol of the manufacturer. Human prostate cancer cells (DU145) (HTB-81, ATCC, Manassas, VA, USA) were cultured at 37°C (in 5% CO_2_) in Eagle’s minimum essential medium (EMEM) (0-2003, ATCC, Manassas, VA, USA) containing 100 U/ml of penicillin and 0.1 mg/ml of streptomycin. The cells were subcultured per the protocol of the manufacturer.

### Cell viability

Human renal mesangial cells or human prostate cancer cells were plated in a 96-well TPP plate and allowed to adhere. Mesangial cells were serum starved for 24 h prior to treatment. The cells were pretreated with PTUPB and incubated for 1 h. Next, sorafenib was added, and the cells were incubated for 48 h. At the end, cell viability was determined using an MTT assay (ab211091, Abcam, Cambridge, MA, USA). The experimental media were aspirated, and the cells were incubated in an MTT reagent solution for 3 h (50 μl of the MTT reagent in 50 μl of FBS-free culture media per well). Next, the MTT reagent solution was aspirated, and the cells were solubilized. The plate was placed on an orbital shaker and mixed at a high setting for 30 min. Absorbance was read using the FLUOstar Omega spectrometer (BMG Labtech Inc., Cary, NC, USA) at 590 nm.

### Proliferation

Mesangial cell proliferation was determined by live cell imaging. Mesangial cells were seeded at 7,500 cells per well in a 96-well TPP plate, allowed to adhere, treated with sorafenib, and imaged over 48 h using an Incucyte system (Sartorius, Göttingen, Germany) configured with a ×4 objective. Cell confluency was calculated as percent area and expressed as fold change relative to the initial confluency at hour 0 using the Fiji image analysis software (ImageJ 1.52s, National Institute of Health, MD, USA).

### Apoptosis

Mesangial cells were seeded on glass coverslips in a 24-well plate and grown to 80%–90% confluency and subsequently treated with sorafenib and/or PTUPB and incubated over 24 h. Apoptosis was measured using the TUNEL assay (C10245, Thermofisher, Waltham, MA, USA) per the instructions of the manufacturer. Cell nuclei were stained using the Hoechst 33342 nuclear dye (1:5,000) provided with the kit. Stained coverslips were visualized at a ×200 magnification with a fluorescence microscope. The analysis was carried out using the Fiji image analysis software (ImageJ 1.52s, National Institute of Health, MD, USA). Total fluorescent signal was measured relative to the number of cells in a field of vision. Analysis was carried out by two observers blinded to the sample identity.

### Statistical analysis

All data are expressed as mean values. GraphPad Prism® Version 4.0 software was utilized to conduct a one-way ANOVA followed by Tukey’s *post-hoc* test to establish statistical significance between the groups (GraphPad Software Inc., La Jolla, CA, USA). Two-tailed unpaired Student’s *t*-test was applied to determine statistical significance between groups. Value of *p* ≤ 0.05 were considered significant.

## Results

### Sorafenib treatment upregulated soluble epoxide hydrolase and cyclooxygenase-2 expression in the kidneys

After 56 days of treatment, sorafenib resulted in an upregulation of mRNA expression of two enzymes metabolizing arachidonic acid metabolites in the kidneys: the soluble epoxide hydrolase enzyme (Ephx2) was upregulated 1.9-fold, and the cycolooxygenase-2 enzyme (COX-2) was upregulated 3.2-fold. (Figure 1B).

### PTUPB mitigated the blood pressure elevation and progression of proteinuria

**FIGURE 2:**
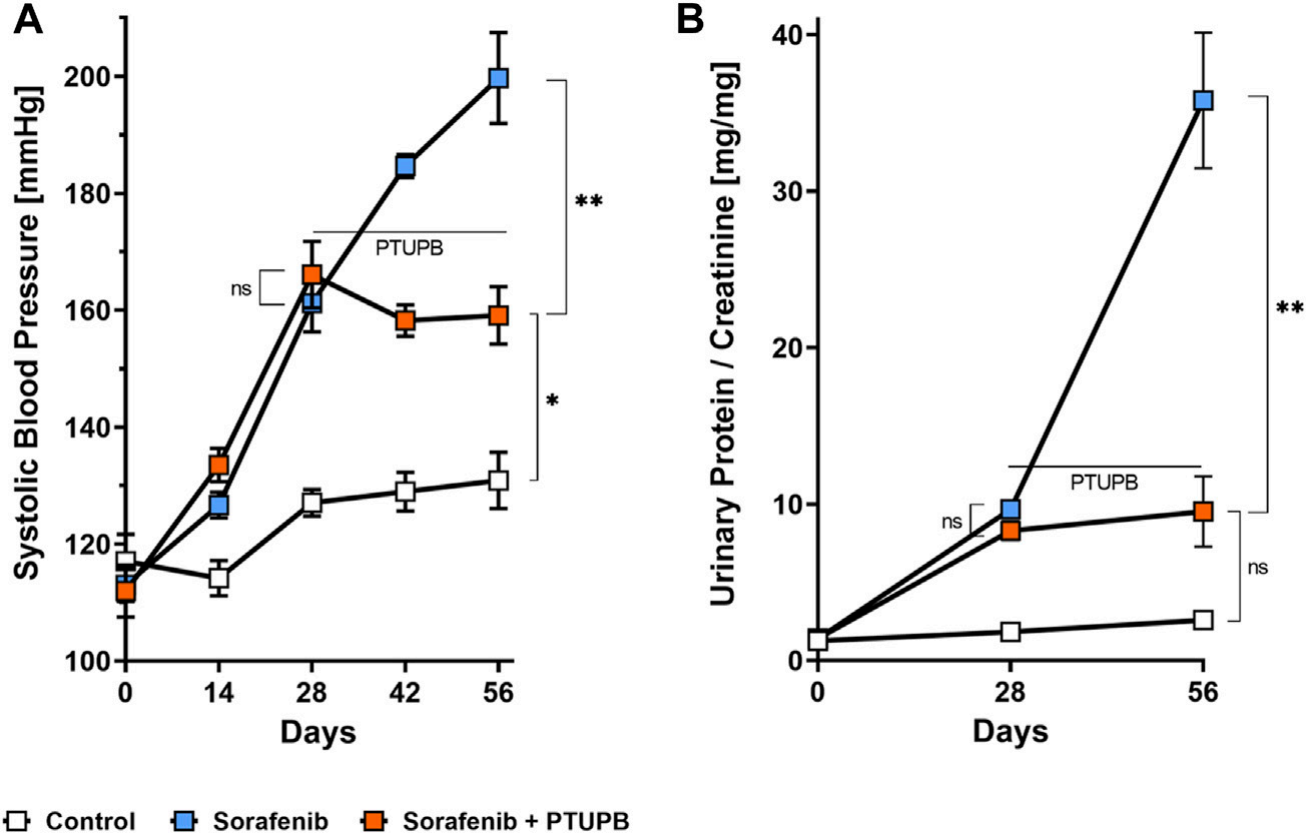
Blood pressure and proteinuria. Hypertension developed in the sorafenib-treated animals (blue trace); the rise in blood pressure was mitigated by 4-(5-phenyl-3-{3-[3-(4-trifluoromethylphenyl)-ureido]-propyl}-pyrazol-1-yl)-benzenesulfonamide (PTUPB) coadministration (orange trace) **(A)**. Proteinuria developed in the sorafenib group (blue trace), which was mitigated by PTUPB (orange trace) **(B)**. The horizontal line marks when PTUPB started being coadministered alongside sorafenib in the PTUPB group (*n* = 4–8 rats/group, average at each time point represents duplicate measures for each individual rat, ns *p* > 0.05, *p ≤ 0.05, **p ≤ 0.01 determined by ANOVA followed by the Tukey *post-hoc* test). Data are reported as connected scatterplots with SEM.

To monitor the overall disease progression and impact on kidney and cardiovascular health, we collected urine samples and measured the blood pressure at designated time points throughout the study. At the outset, the average blood pressure in all three groups was 114 ± 4 mmHg. After the sorafenib treatment started, the blood pressure in the sorafenib-treated and control animals begun to diverge. Halfway into the study (day 28), the blood pressures in the animals that received sorafenib increased by 48 mmHg compared with those of the control group (Figure 2A). PTUPB treatment began on day 28 (Figure 1A). Throughout the remainder of the study, the blood pressure of the animals that were being treated with sorafenib alone continued to increase and ultimately rose by an additional 39 mmHg (day 56). In contrast, blood pressure in the animals of the sorafenib + PTUPB group was decreased by 7 mmHg (Figure 2A).

Urinary protein excretion followed a similar trend. At the beginning protein excretion was low in all animals. Over the course of the study, protein excretion in the control group remained relatively steady. However, the sorafenib-treated animals showed a different trend. On day 28, their urinary protein levels were 4.9-fold higher than the control group. On day 56, animals that received sorafenib alone showed pronounced proteinuria, with urinary protein levels that are 13.9-fold higher than the control group. In contrast, the urinary protein levels in animals that had been cotreated with PTUPB were 73% lower. The data show that the intervention with PTUPB could reverse the progression of sorafenib-induced proteinuria (Figure 2B).

### PTUPB mitigated the progression of renal fibrosis and tubular injury

**FIGURE 3:**
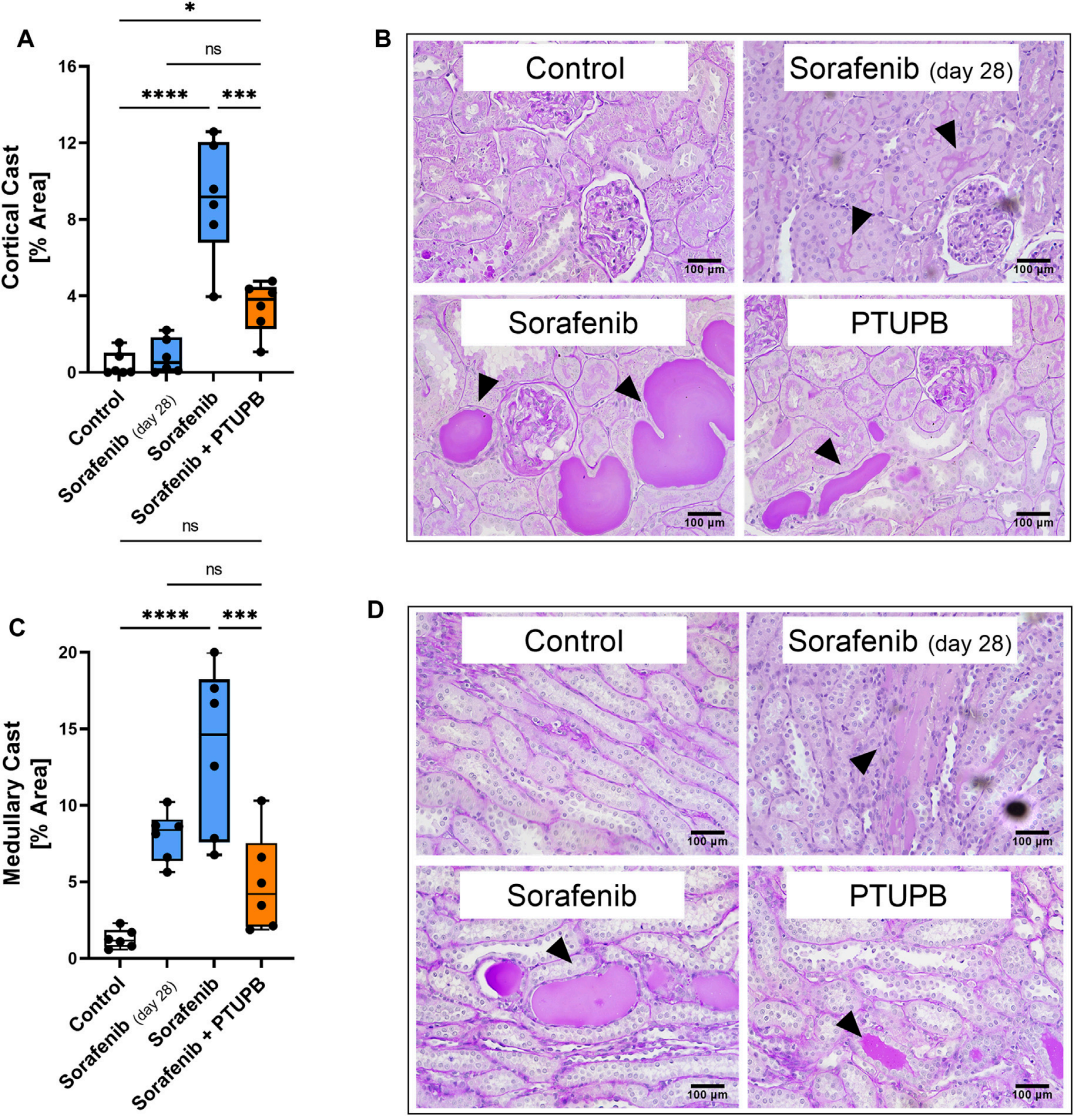
Tubular injury. Sorafenib induced and PTUPB mitigated intratubular cast formation in the cortex **(A)**. Representative images of cortical casts [periodic acid-Schiff (PAS) staining]. Arrowheads point to cast areas. **(B)** Sorafenib induced and PTUPB mitigated intratubular cast formation in the medulla **(C)**. Representative images of cortical casts (PAS staining). Arrowheads point to cast areas. **(D)** (*n* = 6 rats/group. Dots represent the average of duplicate measures for each individual rat., ns *p* > 0.05, *p ≤ 0.05, **p ≤ 0.01, ***p ≤ 0.001, ****p ≤ 0.0001 determined by ANOVA followed by the Tukey *post-hoc* test. Data are reported as box and whisker plots with median, minimum to maximum, and 10 to 90 percentiles.

Tubulopathies appear with kidney injury and indicate disease progression. We assessed the extent of tubular cast formation in the renal cortex and medulla. In general, we found significant amounts of tubular casts after 56 days of sorafenib administration. However, tubular casts covered a significantly lower percent area in the sections of PTUPB-treated animals. We have assessed these changes separately in the cortex and medulla portions of the sections. Although cortical casts were not yet fully evident by day 28 of sorafenib treatment, the beginnings of their formation were visible. After 56 days of sorafenib administration, cortical casts were increased by 21.7-fold compared with the control group. This increase was successfully mitigated by cotreatment with PTUPB, which reduced the cortical casts by 62% compared with the sorafenib group (Figure 3A). Similarly, medullary casts were increased by 6.2-fold after 28 days of sorafenib administration, and by 10.5-fold after 56 days. PTUPB cotreatment lowered the cortical and medullary cast areas by 39% and 64%, respectively (Figure 3B).

**FIGURE 4:**
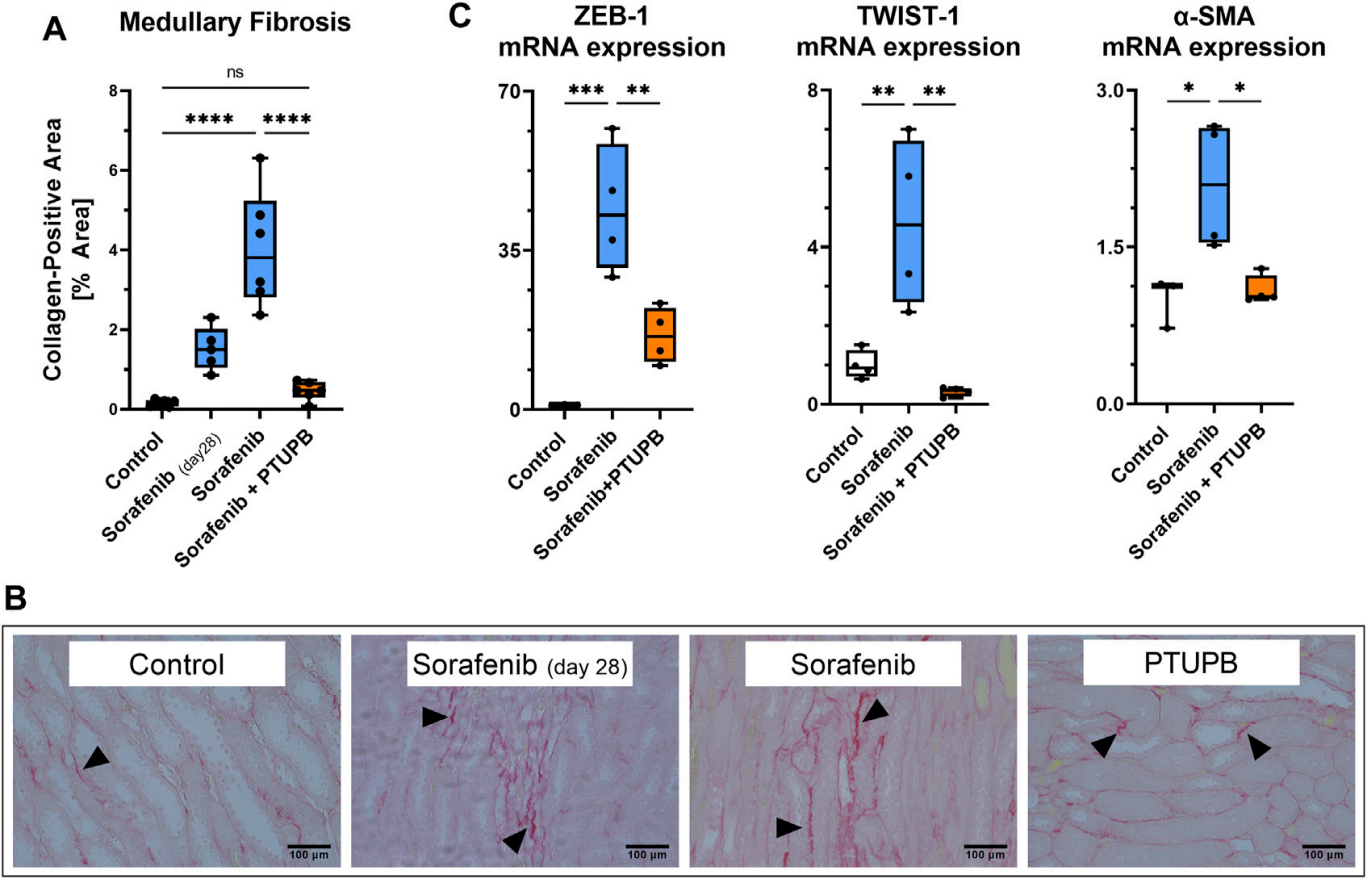
Fibrosis. Fibrotic changes developed in the medulla of sorafenib-treated animals. PTUPB reversed fibrosis progression **(A)**. Representative images of fibrotic changes [picrosirius red (PSR) staining]. Arrowheads point to fibrotic areas. **(B)** Sorafenib treatment elevated ZEB-1 and TWIST-1 (EMT markers) as well as α-SMA (myofibroblast marker). PTUPB mitigated the increase in those markers **(C)**. *n* = 4–6 rats/group. Dots represent the average of duplicate measures for each individual rat. ns *p* > 0.05, *p ≤ 0.05, **p ≤ 0.01, ***p ≤ 0.001, ****p ≤ 0.0001 determined by ANOVA followed by the Tukey *post-hoc* test. Data are reported as box and whisker plots with median, minimum to maximum, and 10 to 90 percentiles.

We next studied the impact of sorafenib and PTUPB on renal fibrosis. Kidney sections were stained with picrosirius ped (PSR) to visualize collagen-positive areas. Kidney fibrotic changes were increased by 9.7-fold after 28 days, and 25.5-fold after 56 days of sorafenib administration. Cotreatment with PTUPB decreased fibrosis by 69% with respect to day 28, and by 88% with respect to day 56; this suggests that PTUPB stopped, or may have even reversed, fibrosis progression (Figure 4B). These fibrotic changes were reflected in epithelial-to-mesenchymal transition (EMT) marker mRNA expression levels. Sorafenib increased ZEB1 expression by 44.1-fold, TWIST expression by 4.6-fold, and α-SMA by 2.1-fold over the control. In contrast, PTUPB treatment reduced ZEB1 expression by 63%, TWIST expression by 93%, and α-SMA expression by 48% compared with the sorafenib group (on day 56). (Figure 4A).

### PTUPB mitigated glomerular injury

**FIGURE 5:**
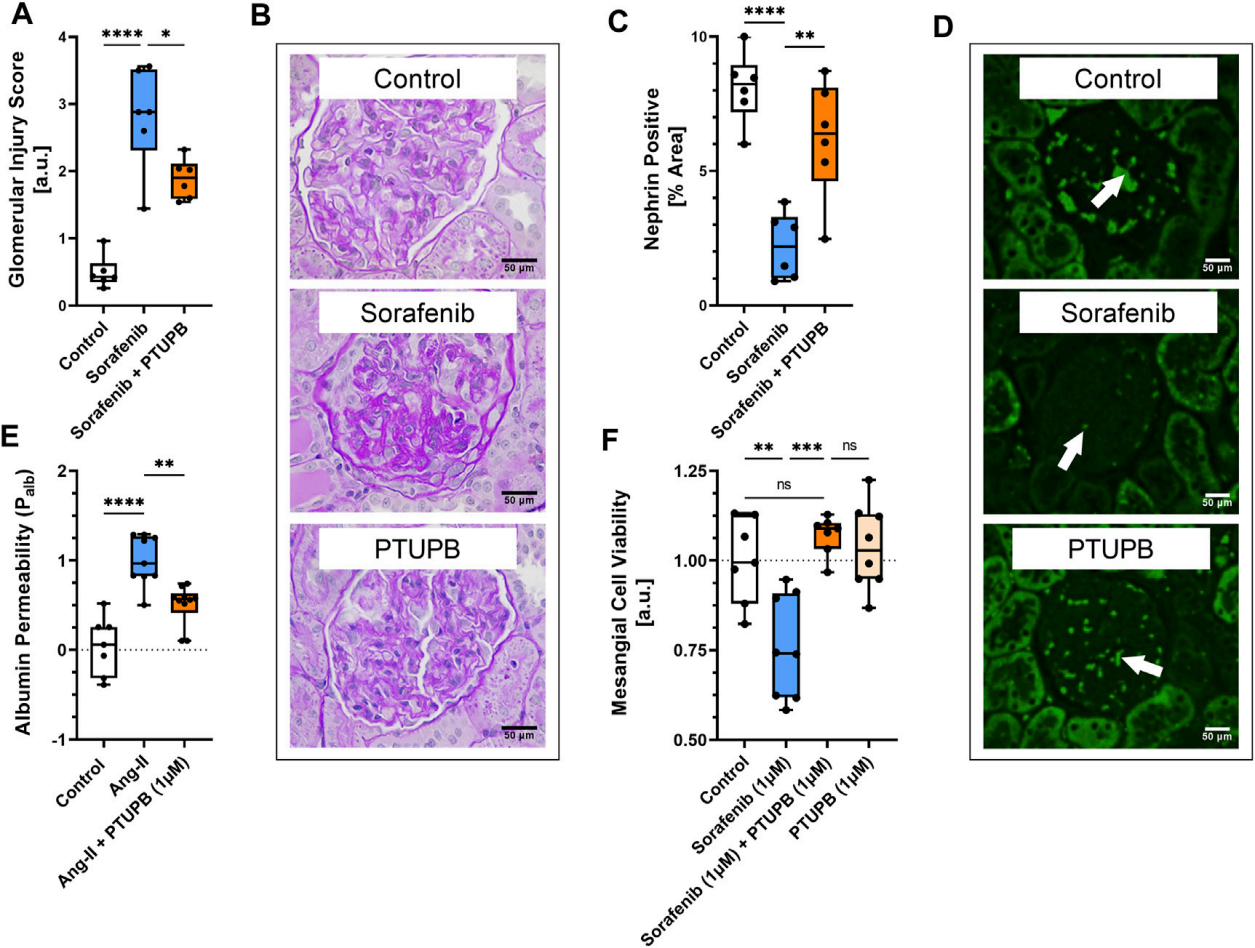
Glomerular injury. Glomerular injury resulted from sorafenib-treatment (blue bar). PTUPB cotreatment improved glomerular histological features (orange bar) **(A)**. Representative images of fibrotic changes (PAS staining) **(B)**. Nephrin loss was evident in sorafenib-treated glomeruli (blue bar). PTUPB mitigated nephrin loss (orange bar) **(C)**. Representative images of nephrin changes in glomeruli (IF, nephrin stain). White arrows point to nephrin-positive areas **(D)**. PTUPB mitigated Ang-II-induced glomerular permeability (orange bar) **(E)**. PTUPB rescued mesangial cells from sorafenib cytotoxicity **(F)**. **(A–D)** *n* = 4–6 rats/group. Dots represent the average of duplicate measures for each individual rat. **(E)** *n* = 7–10 glomeruli from four rats. **(F)** *n* = 7–8. Dots represent the average of duplicate measures for each well. ns *p* > 0.05, *p ≤ 0.05, **p ≤ 0.01, ***p ≤ 0.001, ****p ≤ 0.0001 determined by ANOVA followed by the Tukey *post-hoc* test. Data are reported as box and whisker plots with median, minimum to maximum, and 10 to 90 percentiles.

The extent of glomerular injury was histologically assessed and scored on a 1–4 scale, where a higher number denotes a greater extent of renal injury. After 56 days, the glomerular injury in the sorafenib group was increased by 5.7-fold, which was decreased by cotreatment with PTUPB by 33% (Figures 5A, B). We next determined whether our treatments affected nephrin level changes, as nephrin is a key protein necessary for glomerular health. On day 56, nephrin levels were decreased in the sorafenib group by 73%, and PTUPB cotreatment resulted in a 2.8-fold improvement (Figures 5C, D).

**FIGURE 6:**
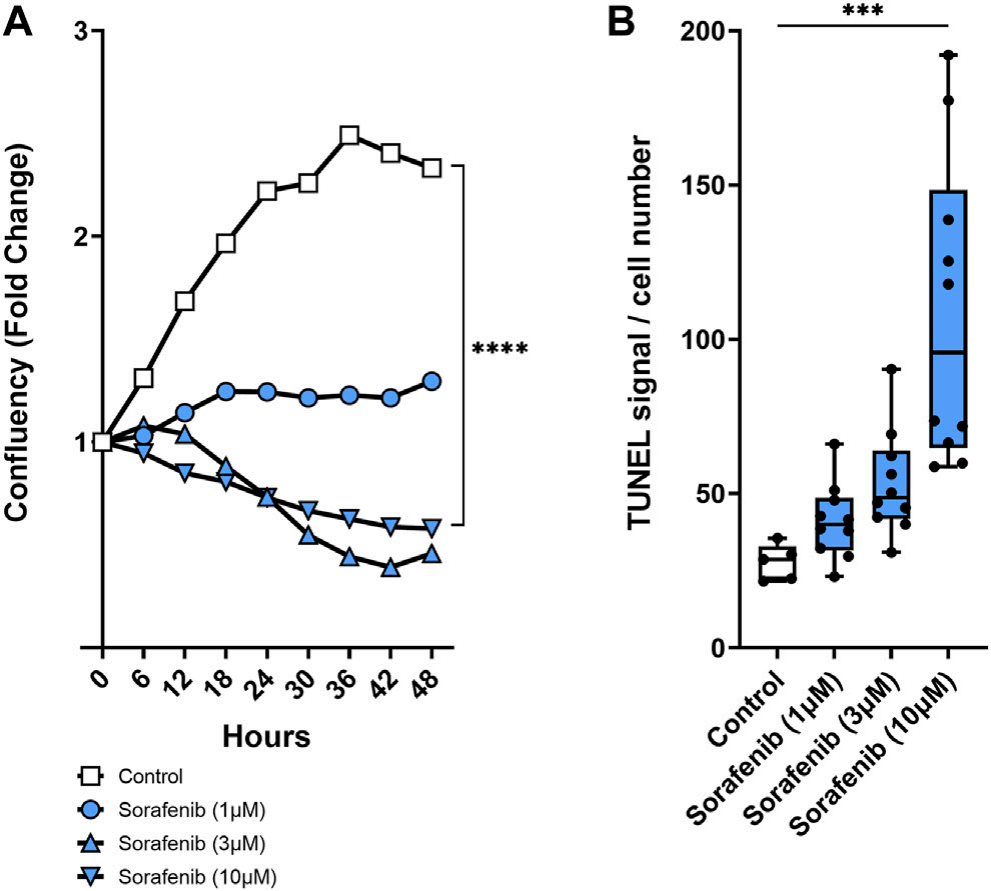
Mesangial cytotoxicity. Sorafenib was cytotoxic to cultured mesangial cells. Sample time–course graph showing decrease in mesangial cell confluency with sorafenib treatment **(A)**. Sorafenib resulted in a dose-dependent increase in mesangial cell apoptosis **(B)**. *n* = 5–10. Dots represent the average of duplicate measures for each well. ***p ≤ 0.001, ****p ≤ 0.0001 determined by ANOVA followed by the Tukey *post-hoc* test. Data are reported as a connected scatterplot and a box and whisker plot with median, minimum to maximum, and 10 to 90 percentiles.

**FIGURE 7:**
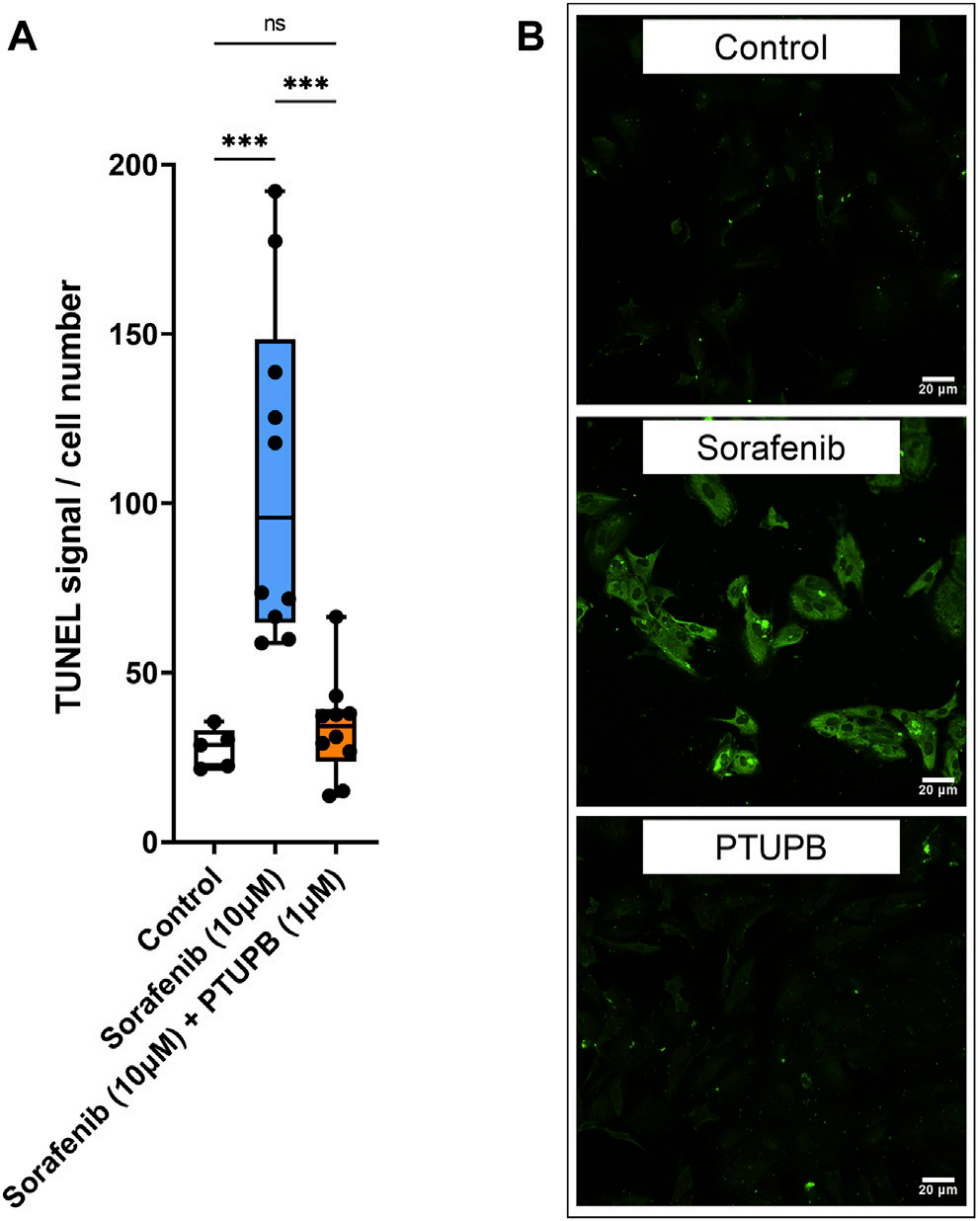
Apoptosis. PTUPB decreased sorafenib-induced apoptosis. Sorafenib treatment elevated apoptotic signal in mesangial cells (orange bar). PTUPB mitigated apoptotic signal in mesangial cells (blue bar) **(A)**. Representative images of TUNEL staining at ×200 (TUNEL signal in green) (*n* = 5–10, dots represent the average of duplicate measures for each well). ns *p* > 0.05, ***p ≤ 0.001 determined by ANOVA followed by the Tukey *post-hoc* test. Data are reported as a box and whisker plot with median, minimum to maximum, and 10 to 90 percentiles.

To see if PTUPB can protect the glomerular filtration barrier itself, we tested its impact on the permeability of isolated glomeruli to albumin *in vitro*. Glomeruli were preincubated with PTUPB, and glomerular permeability was increased with angiotensin II. PTUPB reduced the angiotensin-II-induced glomerular permeability by 51%, which shows that PTUPB can protect the glomerular filtration barrier (Figure 5E). Next, we studied whether PTUPB can impact mesangial cell viability since mesangial cell death, mesangiolysis, is a common feature seen with sorafenib nephrotoxicity. We wanted to test if PTUPB could protect against sorafenib-induced mesangial cytotoxicity. We found that after 48 h, sorafenib decreased mesangial viability by 24% compared with the control, and coincubation with PTUPB increased their viability by 1.4-fold, restoring it back to control levels (Figure 5F). Finally, we evaluated the contribution of apoptosis to sorafenib-induced mesangial cytotoxicity. We confirmed that sorafenib is cytotoxic to cultured mesangial cells (Figure 6A) and determined that sorafenib induced apoptosis in mesangial cells, with the highest dose, 10 μM, increasing apoptotic signal by 3.9-fold (Figure 6B). PTUPB lowered sorafenib-induced mesangial cell apoptosis by 69% (Figures 7A, B). These findings demonstrate direct PTUPB actions at the level of the glomerulus and the mesangial cell to combat sorafenib-induced nephrotoxicity.

### PTUPB does not impair the antitumor activity of sorafenib

**FIGURE 8:**
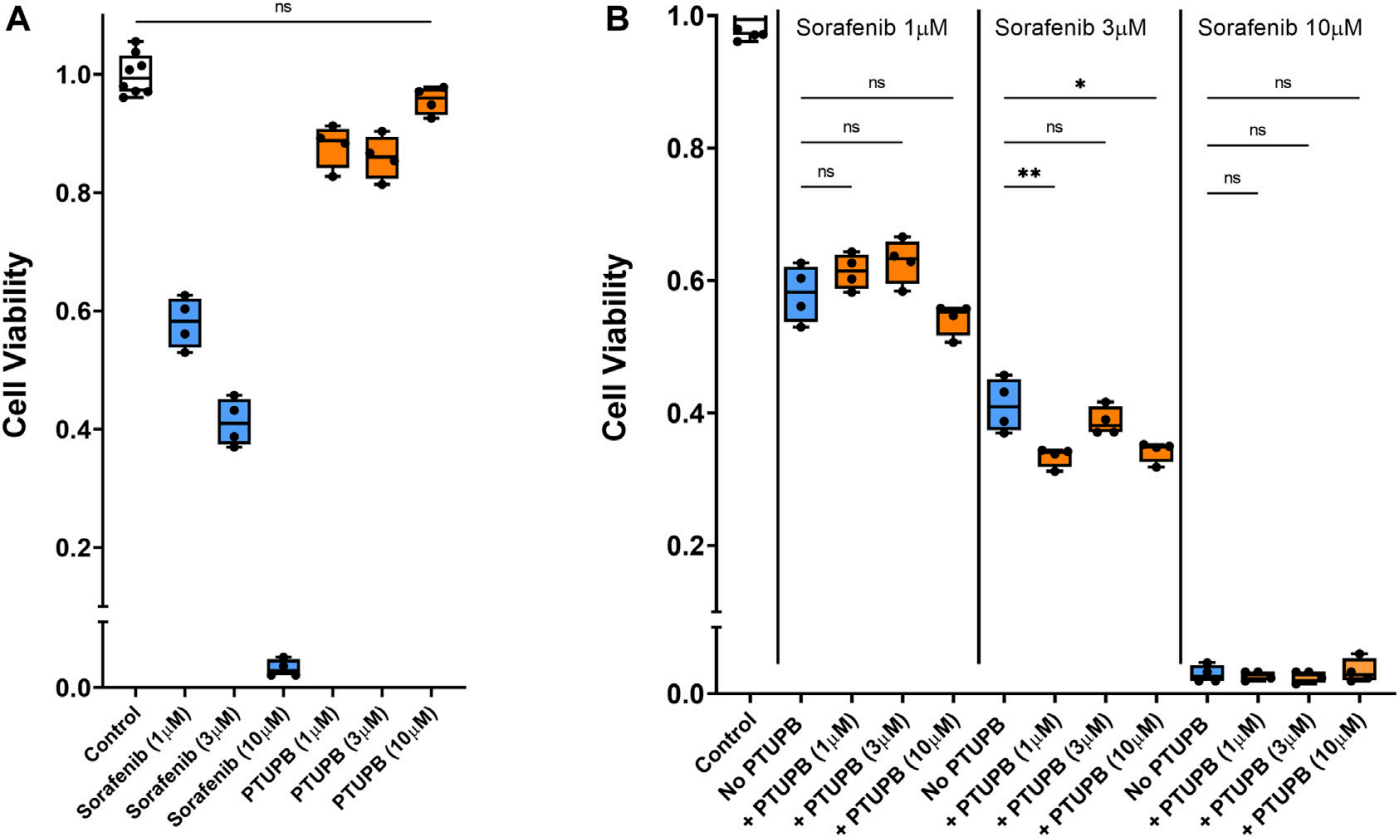
Chemotherapeutic effectiveness. PTUPB did not interfere with the antitumor activity of sorafenib against the DU-145 prostate cancer cells. Sorafenib decreased viability of prostate cancer cells in a dose-dependent fashion, while PTUPB alone lacked cytotoxic effects in these cells **(A)**. In combination with sorafenib, PTUPB did not interfere with the antitumor activity in the DU-145 prostate cancer cells **(B)** (*n* = 4). Dots represent the average of duplicate measures for each well. ns *p* > 0.05, *p ≤ 0.05, **p ≤ 0.01 determined by ANOVA followed by the Tukey *post-hoc* test. Data are reported as box and whisker plots with median, minimum to maximum, and 10 to 90 percentiles.

Finally, we tested whether in combination, PTUPB would interfere with the antitumor activity of sorafenib. To answer this question, human prostate cancer cells were cotreated with sorafenib and PTUPB together. We found that in the 1- to 10-μM range, PTUPB did not adversely affect the antitumor activity of sorafenib (Figure 8B).

## Discussion

Antiangiogenic chemotherapeutics have become a major class of drugs deployed for the treatment of solid tumors (neoplasms). They inhibit the VEGF signaling pathway and neovascularization of tumors, which prevents the blood supply to a tumor, thus, limiting tumor growth (Grothey and Galanis, 2009). Despite their effectiveness as tumor-combating agents, VEGF TKIs can exert a range of adverse effects on cardiovascular and kidney health (Kappers et al., 2009; Perazella, 2012; Estrada et al., 2019; Neves et al., 2020; Dobbin et al., 2021). In the present study, sorafenib caused elevation of kidney sEH and COX-2 enzymes. Previous findings have demonstrated that sEH and COX-2 induction has been linked to inflammation and kidney injury (Imig, 2006). Thus, dual inhibition of sEH and COX-2 enzymes with PTUPB could offer a successful treatment approach. Our findings affirmed it. We found that PTUPB mitigates hypertension in sorafenib-treated animals. The present finding fits the current understanding that sEH inhibition can lower blood pressure (Zhang et al., 2020). This is important as the overall hypertension incidence in patients who receive sorafenib ranges from 6% to 43%, which is a significant clinical limitation for this class of drugs (Wu et al., 2008; Chang et al., 2017; Caletti et al., 2018; Plummer et al., 2019).

Kidney injury is a further complication of VEGF TKI chemotherpy (Izzedine et al., 2010). We found that sorafenib caused extensive proteinuria, and PTUPB successfully mitigated its development, keeping urinary protein levels comparable with those of the control group. Patients who receive sorafenib are at risk for developing proteinuria; a meta-analysis showed that the overall incidence of proteinuria in patients on anti-VEGF therapy is 63% including 6.3% for those treated for renal small cell carcinoma (Izzedine et al., 2010). A meta-analysis showed 12% overall incidence in all grades of proteinuria with sorafenib (Zhang et al., 2014a). Evidence suggests that sEH and COX-2 induction is intimated in inflammation and kidney injury (Imig, 2006; Liu, 2019). Consequently, a genome-wide associate study of 406 subjects found that an sEH polymorphism lowering sEH activity was associated with better outcomes in diabetic nephropathy (Ma et al., 2018). A similar study reported that sEH deletion has been found to mitigate kidney injury caused by streptozotocin in diabetic mice and lessen kidney injury in DOCA-salt hypertension (Manhiani et al., 2009; Elmarakby et al., 2011). At the same time, COX-2 overexpression predisposes podocytes, a major cell type of the glomerular filtration barrier, to mechanical stress injury (Cheng et al., 2007). In addition to targeting VEGF signaling, sorafenib also inhibits sEH (Hwang et al., 2013). However, sorafenib nephrotoxicity persists and is likely due to VEGF signaling actions prevailing at the level of the glomerulus in addition to the increase in kidney COX-2 expression. This suggests that further inhibition of sEH combined with COX-2 inhibition could be beneficial. Indeed, combined sEH and COX-2 inhibition with PTUPB decreased sorafenib-induced nephrotoxicity. Our present finding is consistent with the previous studies using Zucker diabetic rats, where PTUPB was also found to decrease kidney injury and proteinuria (Hye Khan et al., 2016; Khan et al., 2021).

Kidney injury can further manifest in tubular damage (tubulopathies). We found that PTUPB effectively decreased tubular injury both in the renal cortex and medulla. A common manifestation of various kinds of kidney injury is fibrosis, and VEGF inhibition has been found to exacerbate fibrosis in other organ systems such as the lungs (Partovian et al., 2000; Tuleta and Frangogiannis, 2021). We found that PTUPB halted the progression of renal fibrosis caused by sorafenib. Furthermore, the extent of fibrotic damage was less than at the outset of the treatment (day 28), which suggests that PTUPB may have reversed some of the fibrotic damage. This is also consistent with our previous work in the diabetic nephropathy model where we found that an 8-week treatment with PTUPB decreased fibrosis in Zucker diabetic rats and lowered it to control levels (Hye Khan et al., 2016). The effects of PTUPB on tissue fibrosis have been studied in the context of pulmonary fibrosis and non-alcoholic fatty liver disease. Zhang et al. (2020) found that PTUPB pretreatment decreased fibrosis in the lungs of bleomycin-treated mice. Remarkably, PTUPB was effective for treating fibrosis even when introduced at a stage when fibrotic changes were mature; much like in the present study, the fibrotic changes were reversed. Zhang et al. (2020) presented evidence that these effects of PTUPB on fibrosis likely were mediated by inhibition of senescence. Sun et al. (2020) found that liver fibrosis induced by a high-fat diet was significantly diminished when mice received PTUPB over the course of 12 weeks. In the kidneys, renal tubular epithelial cells can transform into activated fibroblasts, called myofibroblasts, which highly express α-SMA. Myofibroblasts then secrete extracellular proteins such as collagens and fibronectin (Fragiadaki and Mason, 2011). This transformation process is referred to as EMT. TWIST and ZEB1 are transcription factors that promote EMT (Vandewalle et al., 2009; Fragiadaki and Mason, 2011; Wang et al., 2016; Sheng and Zhuang, 2020). Notably, we found that PTUPB lowered EMT—it downregulated 1) the transcriptional activators of EMT, TWIST and ZEB1, and 2) a myofibroblast marker, α-SMA. In the lung, Zhang et al. observed a similar decrease in α-SMA levels with PTUPB treatment (Zhang et al., 2020). The same effect of lowering α-SMA protein expression in the liver was reported by Sun et al. (2020). Furthermore, PTUPB was found to inhibit glioblastoma growth through a mechanism that involved a drastic downregulation of ZEB1 and which the authors believed was linked to PTUPB inhibiting HMMR/SOX-2 signaling (Li et al., 2017). In summary, it appears that a plausible mechanism by which PTUPB mitigates renal damage (tubular damage and fibrosis) involves blocking the transformation of renal epithelial tubular cells into myofibroblasts through EMT.

Glomerular health is vital to renal function. We found that PTUPB mitigated the otherwise extensive glomerular damage features caused by sorafenib. The glomerulus is the part of a nephron where plasma filtration takes place. This is made possible by the glomerular filtration barrier that allows for retaining elements in the plasma (and keeping them out of the filtrate). At least one likely mechanism through which PTUPB fortifies glomeruli against injury is by preventing podocyte nephrin loss. Nephrin is essential to glomerular health. Expressed by podocytes, it participates in forming the slit diaphragm, a structure that supports the glomerular filtration barrier functional integrity (Yu et al., 2018). Nephrin loss ultimately leads to podocyte effacement and proteinuria (Faul, 2014; Garg, 2018). We found that sorafenib caused significant loss of nephrin in the glomeruli. This is consistent with other reports indicating that VEGF signaling inhibition results in nephrin loss (Izzedine et al., 2010; Lankhorst et al., 2015; Terrasse, 2015). Remarkably, PTUPB treatment prevented sorafenib-induced nephrin loss and restored it to levels similar to those of the control. We further confirmed that PTUPB protected the glomerular filtration barrier from injury in isolated glomeruli. This is an important finding for its clinical corollaries. Damage to the glomerular filtration barrier manifests in proteinuria (Yu et al., 2018), which is commonly reported in patients receiving VEGF TKI chemotherapy and has even been reported following intravitreal VEGF TKI delivery (Patel et al., 2008; Estrada et al., 2019; Hanna et al., 2019). To our knowledge, this is the first study reporting the effects of PTUPB on the glomerular filtration barrier.

While the exact mechanism through which inhibition of VEGF signaling leads to glomerular damage remains unknown, it likely involves an interplay between various effects on the cross-talk among glomerular cells (Estrada et al., 2019). Mesangial cells are an important glomerular constituent, and their death (mesangiolysis) has been a reported feature of sorafenib nephrotoxicity (Overkleeft et al., 2009; Schlöndorff and Banas, 2009). Here, we show that sorafenib treatment caused mesangial cell death*.* This effect has not been extensively studied elsewhere, but it is of importance since mesangial cells support the structural and functional integrity of the glomerulus (Schlöndorff and Banas, 2009). The survival of mesangial cells is dependent on VEGF singling. For example, the diminished VEGF-A secretion by podocytes can lead to mesangiolysis. In addition, mesangial cells also produce their own VEGF, which further regulates their growth through an autocrine mechanism (Tahara et al., 2011). Sorafenib targets VEGFR-2 and VEGFR-3. Human renal mesangial cells express VEGFR-1 and VEGFR-2 (Thomas et al., 2000). Thus, sorafenib likely causes mesangiolysis by blocking singling through VEGFR-2. Importantly, PTUPB showed potency in protecting mesangial cells from this sorafenib-induced cytotoxicity. This hints at the possibility that affecting eicosanoid signaling through dual sEH/COX-2 inhibition restores some of the downstream effects of VEGFR-2 signaling or activates cellular pathways that offset the negative effects of VEGFR-2 blockage. Together, the effects on nephrin, glomerular filtration barrier, and mesangial cells suggest that the ability of PTUPB to restore glomerular health is independent of blood pressure lowering. If so, dual sEH/COX-2 inhibition by PTUPB could be a viable treatment strategy for VEGF-TKI-induced renal damage.

Intriguingly, PTUPB has been found to inhibit tumor growth and combat cancer (Li et al., 2017; Wang et al., 2018). These studies have evaluated ovarian tumor growth, glioblastoma growth, and Lewis lung carcinoma growth and metastasis (Zhang et al., 2014b; Li et al., 2017; Gartung et al., 2019). In addition, PTUPB has been found to potentiate the cisplatin anticancer activity in a mouse xenograft model of bladder cancer (Wang et al., 2018). We tested PTUPB alone and in combination with sorafenib on prostate tumor cells. Our data demonstrate that PTUPB did not lead to prostate tumor cell death or significantly enhance the ability of sorafenib to cause tumor cell death. Importantly, PTUPB did not interfere with the ability of sorafenib to cause prostate tumor cell death. These findings provide evidence that PTUPB can prevent sorafenib-induced nephrotoxicity without interfering with sorafenib-mediated anticancer actions.

## Conclusion

Since their inception in 1990, VEGF TKIs have been proven effective against several cancers. Our ability to harness their potential in killing neoplasm cells, however, is limited by their serious adverse effects on cardiovascular and kidney health, which sometimes may force discontinuation of an otherwise effective therapy (Venkatachalam et al., 2010; Grenon, 2013; Porta et al., 2015; Neves et al., 2020). Kidney damage is especially problematic because it can progress to end-stage renal disease if not treated. This provides a strong rationale for developing a combination approach that can protect from the progressive VEGF-TKI-induced kidney damage. Data presented in this study suggest that dual sEH/COX-2 inhibition with PTUPB could be effective as such strategy because it successfully mitigated the nephrotoxic effects of a VEGF TKI, sorafenib. We show that PUTPB reduced hypertension and proteinuria, mitigated tubular and fibrotic injury, and improved glomerular health. Our *in vitro* glomerular and cultured mesangial cell data further support our understanding of VEGF signaling and glomerular biology, and the interplay between VEGF and eicosanoid signaling pathways. Additionally, PTUPB does not interfere with the anticancer actions of sorafenib. Together, the results show that PTUPB could be an effective therapeutic agent against VEGF TKI nephrotoxicity and perhaps also other conditions resulting in glomerular injury. Further investigation is warranted.

## Data Availability

The raw data supporting the conclusion of this article will be made available by the authors, without undue reservation.
